# SGNet: A Structure-Guided Network with Dual-Domain Boundary Enhancement and Semantic Fusion for Skin Lesion Segmentation

**DOI:** 10.3390/s25154652

**Published:** 2025-07-27

**Authors:** Haijiao Yun, Qingyu Du, Ziqing Han, Mingjing Li, Le Yang, Xinyang Liu, Chao Wang, Weitian Ma

**Affiliations:** 1 School of Electronic Information Engineering, Changchun University, Changchun 130022, China; 230401175@mails.ccu.edu.cn (Q.D.); 230401178@mails.ccu.edu.cn (Z.H.); limj@ccu.edu.cn (M.L.); 230402201@mails.ccu.edu.cn (L.Y.); 230402186@mails.ccu.edu.cn (X.L.); 240401194@mails.ccu.edu.cn (C.W.); 240401180@mails.ccu.edu.cn (W.M.); 2 School of the Graduate, Changchun University, Changchun 130022, China

**Keywords:** computer-aided diagnosis, structure guidance, feature fusion, Visual Mamba

## Abstract

Segmentation of skin lesions in dermoscopic images is critical for the accurate diagnosis of skin cancers, particularly malignant melanoma, yet it is hindered by irregular lesion shapes, blurred boundaries, low contrast, and artifacts, such as hair interference. Conventional deep learning methods, typically based on UNet or Transformer architectures, often face limitations in regard to fully exploiting lesion features and incur high computational costs, compromising precise lesion delineation. To overcome these challenges, we propose SGNet, a structure-guided network, integrating a hybrid CNN–Mamba framework for robust skin lesion segmentation. The SGNet employs the Visual Mamba (VMamba) encoder to efficiently extract multi-scale features, followed by the Dual-Domain Boundary Enhancer (DDBE), which refines boundary representations and suppresses noise through spatial and frequency-domain processing. The Semantic-Texture Fusion Unit (STFU) adaptively integrates low-level texture with high-level semantic features, while the Structure-Aware Guidance Module (SAGM) generates coarse segmentation maps to provide global structural guidance. The Guided Multi-Scale Refiner (GMSR) further optimizes boundary details through a multi-scale semantic attention mechanism. Comprehensive experiments based on the ISIC2017, ISIC2018, and PH2 datasets demonstrate SGNet’s superior performance, with average improvements of 3.30% in terms of the mean Intersection over Union (mIoU) value and 1.77% in regard to the Dice Similarity Coefficient (DSC) compared to state-of-the-art methods. Ablation studies confirm the effectiveness of each component, highlighting SGNet’s exceptional accuracy and robust generalization for computer-aided dermatological diagnosis.

## 1. Introduction

**Figure 1:**
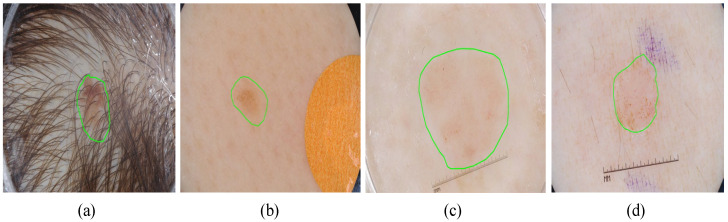
Dermoscopy lesion images: (**a**) lesion with hair interference, (**b**) small lesion with blurred boundaries, (**c**) large lesion with blurred boundaries, and (**d**) low-contrast lesion.

Melanoma, the deadliest skin cancer, originates from epidermal melanocytes and is often refractory and metastatic. Early detection is critical as it enables successful surgical treatment, whereas advanced disease is highly aggressive, rapidly metastasizes, and has a low survival rate [1]. According to the 2025 Global Cancer Statistics [2], it is estimated that there will be 104,960 new cases of melanoma in the United States in 2025. Computer-aided diagnostic technologies for skin cancer can help alleviate physicians’ workload by quickly and accurately localizing skin lesions. This enables doctors to better diagnose skin lesions and implement targeted treatment plans [3]. However, skin image segmentation faces several challenges, including low contrast [4], irregular boundaries, hair interference, and blurred edges, as shown in Figure 1.

Existing skin lesion image segmentation methods can be broadly divided into two categories: traditional image processing methods and deep learning methods. The former includes clustering-based image segmentation [5], region growing [6], and edge detection algorithms [7], but these methods have limited effectiveness when handling complex structures and low-contrast images. With the development of computer vision technology, deep neural networks have made significant progress in skin lesion image segmentation and have become the mainstream approach in current research on skin lesion image segmentation. The early UNet [8] adopted a classic symmetric encoder–decoder architecture, achieving end-to-end medical image segmentation and being widely cited and extended. To further enhance the network’s ability to model multi-scale semantics and boundary information, numerous methods have been proposed. For instance, Ce-net [9] introduced dilated convolutions and context extraction modules to expand the receptive field and capture richer contextual information; MTL-CNN [10] employs a multi-task learning framework, with a shared encoder and parallel decoders, effectively improving skin lesion segmentation and classification performance; Attu-net [11] and ResUNet++ [12] incorporated attention mechanisms and residual connections to enhance the focus on key regions; TransUNet [13] combined CNN and Transformer architectures to model long-range dependencies, while preserving local details; MFEFNet [14] introduced multi-scale feature enhancement and fusion strategies, combined with attention mechanisms, to improve image segmentation accuracy; SwinUnet [15] introduced pure Transformer structures to medical image segmentation, further advancing the semantic modeling capabilities; UNet++ [16] redesigned the skip connections by introducing nested and dense connection structures, achieving more effective feature aggregation; FAT-Net [17] integrates a CNN–Transformer dual encoder with adaptive feature fusion and memory-efficient decoding for precise skin lesion segmentation, enhancing global context capture; nnU-Net [18] introduced a self-configuring framework that automatically adapts the preprocessing, architecture, training, and inference for each dataset, achieving strong generalization across diverse medical segmentation tasks; Li et al. [19] proposed a feature enhancement and interaction Transformer module to improve feature extraction and capture global contextual information, addressing the limitations of traditional deep convolutional neural networks in capturing global information; DMSA-Unet [20] enhances the performance of UNet for medical image segmentation through a dual multi-scale attention mechanism, integrating the strengths of CNNs and Transformers to optimize global and local feature extraction; Sharp U-Net [21] employs Sharp blocks with depthwise separable convolutions and spatial sharpening filters to process encoder feature maps before decoder fusion, enhancing feature extraction, reducing semantic gaps, and eliminating early training artifacts.

Deep learning models that are tailored for skin lesion segmentation have emerged to address challenges like blurry boundaries, low contrast, and hair interference. MSF-Net [22] adopted a multi-scale fusion strategy and boundary attention mechanism, significantly improving the capture of fine-grained edges; DermoNet [23] incorporated dermatological features into the decoding process, guiding the network to learn boundary regions and structural texture distributions of lesions; CA-Net [24] introduced channel and spatial attention mechanisms (Dual Attention), improving the model’s sensitivity to different types of lesion regions and achieving stable performance in skin lesion image segmentation tasks; MHorUNet [25] leveraged recursive gated convolutions and high-order spatial interactions to construct a high-order spatial interaction mechanism, combined with multi-level and multi-scale fusion strategies, enabling accurate segmentation, even when dealing with complex backgrounds and blurred boundaries; LCAUnet [26] introduced a dual-branch architecture with local cross-attention and multi-scale prior fusion, enhancing boundary–body feature integration and improving segmentation accuracy in regard to irregular and blurred lesions.

Despite the progress made in dermoscopic image processing, these methods struggle with complex lesions that exhibit structural ambiguity, broken boundaries, or high regional heterogeneity. On the one hand, some models incorporate attention mechanisms and boundary-guided modules, but struggle to balance boundary localization accuracy with overall computational efficiency. On the other hand, while certain multi-scale fusion methods achieve high-level semantic consistency, they often fall short in regard to coupling shallow texture with deep semantics, leading to edge discontinuity or the omission of small targets, which hinders their practical deployment in clinical scenarios.

To address these challenges, we propose SGNet, a skin lesion segmentation network that enhances the accuracy and robustness of lesion segmentation in complex dermoscopic images through multi-scale feature extraction and boundary optimization. Experiments demonstrate SGNet’s superior performance and generalization across multiple datasets.

(1) We propose SGNet, a hybrid model combining convolutional neural networks (CNNs) and the Mamba structure, consisting of five key modules: VMamba, DDBE, STFU, SAGM, and GMSR, for the precise segmentation of skin lesion images.
(2) The VMamba encoder extracts multi-level features from the input image. To suppress noise and enhance boundary cues, the DDBE refines low-level features via spatial and frequency-domain operations. The STFU then combines these with high-level semantics through adaptive fusion, producing multi-scale representations that support subsequent lesion localization and refinement.
(3) To address lesion localization ambiguities, the SAGM aggregates the STFU features to generate a coarse lesion segmentation map, guiding subsequent refinement. The GMSR enhances boundary details through multi-scale convolutions and a semantic-guided attention mechanism, improving the representation of irregular lesion morphologies.
(4) We evaluated SGNet based on skin lesion datasets, ISIC2017 [27], ISIC2018 [28], and PH2 [29], and compared it with state-of-the-art segmentation methods. The experimental results demonstrate that SGNet achieves outstanding segmentation performance.

## 2. Related Work

In recent years, with the success of the Transformer architecture in vision tasks, its application in the field of medical image segmentation has gradually expanded. For example, CTC-Net [30] combines CNNs and the Swin Transformer, enhancing medical image segmentation performance through a feature fusion module and a single Transformer decoder. Medical Transformer [31] involved the proposal of a medical image segmentation method, based on gated axial attention and Transformer architecture. These methods excel at segmenting large-scale lesion regions, but face challenges due to the high computational and memory demands related to high-resolution images and their limited ability to model fine boundary details and local textures, particularly in complex dermoscopic images, with blurred boundaries and low contrast.

To address the above challenges, Mamba [32], a novel Selective State Space Model, was proposed. It features linear time complexity, a dynamic input control capability, and strong long-range modeling ability. Based on state space theory, Mamba utilizes continuous–discrete mapping to efficiently model sequential information and learns long-term dependencies through a state update function, balancing modeling capacity and computational efficiency. Based on this, the visual state space model, VMamba [33], was proposed. In regard to image tasks, it achieves non-causal modeling through image patch serialization and a cross-scanning strategy, incorporating the Mamba structure into the image-encoding process, and significantly improving the model’s performance and computational efficiency in image classification and semantic segmentation tasks.

As Mamba continues to mature in the visual domain, researchers have begun to explore integrating its structure into medical image segmentation tasks, achieving a series of results, especially in skin lesion image processing. U-Mamba [34] embeds the Mamba module into the nnUNet encoder, leveraging its long-range modeling capability to enhance the understanding of complex structures and improve the modeling of semantic continuity between tissues; VM-UNet [35] constructs a fully Mamba-based segmentation network, achieving a balance between efficient inference and good accuracy in skin lesion tasks; the subsequent VM-UNetV2 [36] further integrates the Semantic-Detail Injection mechanism to enhance the alignment between shallow texture information and deep semantics, significantly improving boundary representation and segmentation consistency; SegMamba [37] involves the design of a tri-directional Mamba path structure for 3D medical images, modeling the dependencies among voxel sequences from axial, sagittal, and coronal planes, achieving excellent 3D segmentation performance; SkinMamba [38], based on frequency guidance and state space modeling mechanisms, demonstrates stronger boundary awareness and discriminative capability in low-contrast and blurred-boundary images. In this paper, we introduce a new hybrid CNN and Mamba architecture, specially designed for the precise segmentation of skin lesion images, capable of achieving accurate boundary localization, even in complex scenarios.

## 3. Method

**Figure 2:**
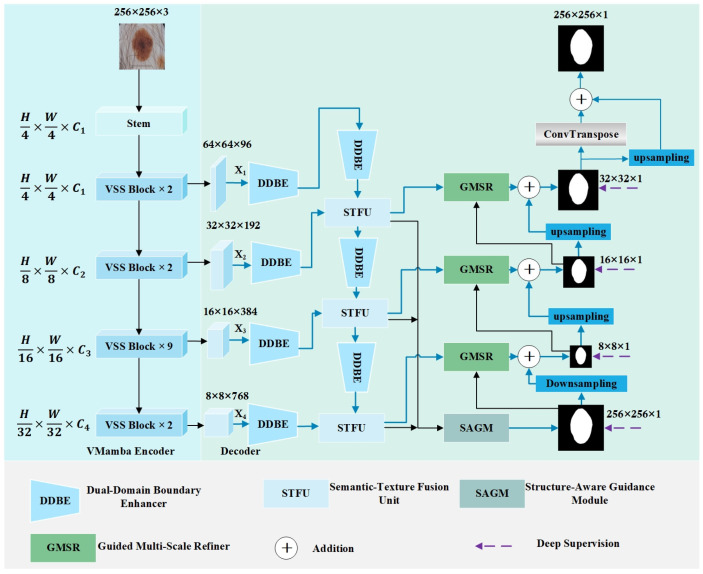
The network structure of SGNet.

The SGNet architecture, as shown in Figure 2, comprises five key modules, namely VMamba, DDBE, STFU, GMSR, and SAGM, designed to enhance skin lesion segmentation. The VMamba encoder extracts high-level and low-level features from the input image. The DDBE enhances the boundary localization of multi-scale features by integrating spatial and frequency-domain processing, effectively suppressing noise artifacts, such as hair interference, to improve lesion segmentation accuracy. The STFU integrates features from different layers to capture multi-scale information, thereby improving feature extraction. The SAGM aggregates features from different levels to generate a coarse segmentation map, providing global guidance. The GMSR enhances the boundary details of features at different levels based on the global guidance provided by the coarse segmentation map generated by the SAGM, thereby improving the representation of irregular lesion morphologies. Each module will be elaborated in the following sections.

### 3.1. VMamba Encoder

CNNs [39] and Vision Transformers (ViTs) [40,41] are two commonly used backbone models for visual feature representation. CNNs are renowned for their excellent scalability, with computational complexity that increases linearly with image resolution, whereas ViTs exhibit stronger modeling capabilities, due to their global receptive field and dynamic weights, albeit at the cost of quadratic complexity growth. VMamba, building on the strengths of both, significantly improves the model’s computational efficiency. By introducing the cross-scanning module, it effectively addresses the issue of direction sensitivity, transforming non-causal visual images into ordered block sequences. This model achieves linear complexity without sacrificing the global receptive field, showing outstanding potential and application prospects in regard to various visual perception tasks. We use VMamba as the encoder and the backbone for feature extraction. A given input image is denoted as $X\in R^{H\times W\times 3}$, where the three channels correspond to the red (R), green (G), and blue (B) channels of the RGB color space. These channels capture the color information of skin lesion images, enabling the model to extract visual features that are critical for lesion segmentation. The VMamba encoder extracts features at different levels, denoted as $X_{i}\in R^{\frac{H}{2^{i+1}}\times \frac{W}{2^{i+1}}\times C_{i}}$, where $C_{i}\in \left(96,192,384,768\right)$ and $i\in \left(1,2,3,4\right)$. In regard to these features, $X_{1}$ provides precise boundary information on the target, while $X_{2},X_{3},$ and $X_{4}$ contain higher-level semantic information.

### 3.2. Dual-Domain Boundary Enhancer

**Figure 3:**
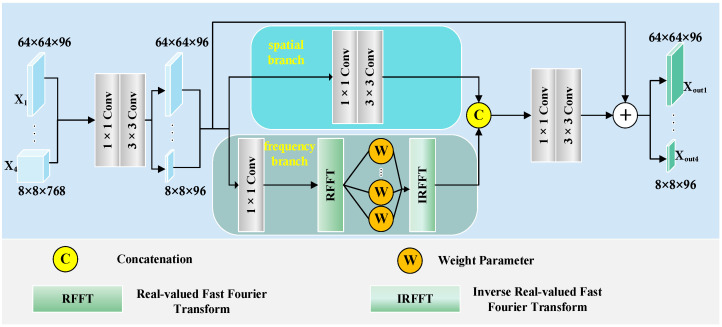
Dual-Domain Boundary Enhancer (DDBE).

In skin lesion segmentation tasks, lesion boundaries are often ambiguous, irregular in shape, and exhibit low contrast with surrounding tissues. To enhance the model’s sensitivity to boundary cues and structural consistency, we designed a DDBE that incorporates a dual-branch design, integrating spatial detail modeling and frequency-domain context reasoning, as shown in Figure 3.

Given the multi-scale features $X_{i},i\in \left(1,2,3,4\right)$ extracted by the VMamba encoder, each feature map is first processed through a 1 × 1 convolution to unify the channel dimension to 96, followed by batch normalization and GELU activation, resulting in the intermediate feature representation $X_{init}\in R^{H_{i}\times W_{i}\times 96}$. This uniform channel configuration effectively reduces the computational cost, while preserving its discriminative representation power, facilitating efficient integration with subsequent modules.

In this module, $X_{init}$ is split into two parallel branches. In the spatial branch, a 3 × 3 convolution is applied to extract local texture and edge-aware details, producing spatial features $X_{spatial}$. In regard to the frequency branch, we apply a 2D Real-valued Fast Fourier Transform (RFFT) to project the input $X_{f}\in R^{H\times W\times 48}$ into the frequency domain. This process is shown in Equation (1).

(1) $${\hat{x}}_{f}\left(u,v\right)=\sum_{x=0}^{H-1}\sum_{y=0}^{W-1}X_{f}\left(x,y\right)\cdot e^{-2\pi i\left(\frac{ux}{H}+\frac{vy}{W}\right)}$$

where ${\hat{x}}_{f}\left(u,v\right)\in C^{H\times \left(\frac{W}{2}+1\right)}$ represents the complex-valued frequency spectrum of the input feature map $X_{f}\left(x,y\right)$, which has 48 channels. The coordinates $\left(u,v\right)$ and $\left(x,y\right)$ denote the frequency and spatial domains, respectively.

To enable adaptive frequency modeling, we introduce a learnable complex-valued weight map $W_{f}\left(u,v\right)$, which modulates the frequency response. This process is shown in Equation (2).

(2) $${\hat{x}’}_{f}\left(u,v\right)={\hat{x}}_{f}\left(u,v\right)\cdot W_{f}\left(u,v\right)$$

where ${\hat{x}’}_{f}\left(u,v\right)$ is the modulated frequency spectrum, and ${\hat{X}}_{f}\left(u,v\right)$ is the original frequency spectrum obtained from the RFFT.

The modulated spectrum is then transformed back into the spatial domain using the inverse FFT (IRFFT). This process is shown in Equation (3).

(3) $$X_{\mathrm{freq}}\left(x,y\right)=\frac{1}{HW}\sum_{u=0}^{H-1}\sum_{v=0}^{W-1}{\hat{x}’}_{f}\left(u,v\right)\cdot e^{2\pi i\left(\frac{ux}{H}+\frac{vy}{W}\right)}$$

where $X_{\mathrm{freq}}\left(x,y\right)$ is the reconstructed feature map in the spatial domain, $e^{2\pi i\left(.\right)}$ is the complex exponential kernel used in the inverse Fourier transform, and $\frac{1}{HW}$ is the normalization factor.

The frequency-domain branch, using RFFT and learnable complex-valued weight modulation, enhances the global structure modeling and shape continuity, complementing the spatial branch focus on local boundary details. The outputs are concatenated along the channel dimension, processed via a fusion block with grouped convolutions and nonlinear activations, and combined through a residual connection to the yield $X_{out}\in R^{H_{i}\times W_{i}\times 96}$. This module enhances the lesion boundary localization capability by jointly modeling fine-grained edges and long-range dependencies.

### 3.3. Semantic-Texture Fusion Unit

**Figure 4:**
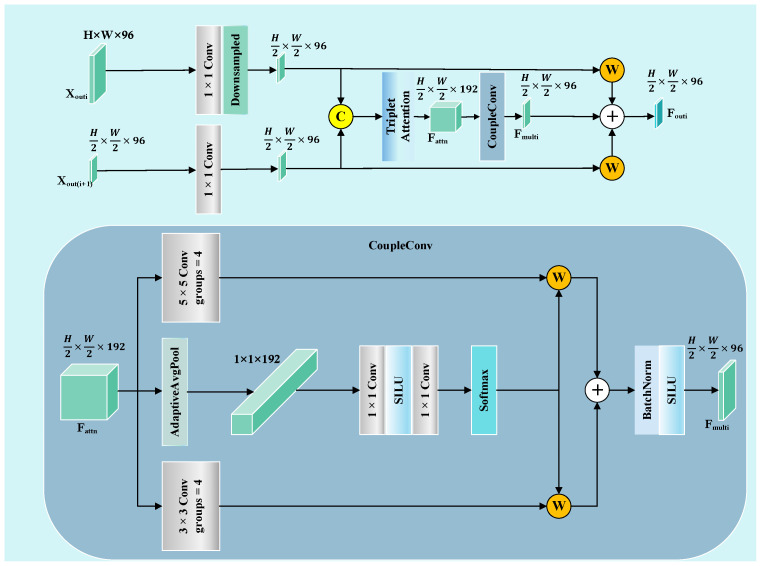
Semantic-Texture Fusion Unit (STFU).

During skin lesion segmentation, disparities in spatial scale and semantic levels between shallow and deep features can lead to inaccurate lesion representation during direct fusion, impairing segmentation performance. To address this issue, we designed the STFU, as shown in Figure 4, to effectively integrate shallow features rich in boundary and texture details with deep features possessing strong semantic discrimination capability, thereby improving segmentation.

To effectively integrate adjacent feature representations $X_{{\mathrm{out}}_{i}}$ and $X_{{\mathrm{out}}_{i+1}},\;i\in \left(1,2,3\right)$, the STFU first applies nonlinear transformation and spatial alignment to both inputs. This process is shown in Equation (4).

(4) $$X_{i}^{’}=\phi \left(X_{{\mathrm{out}}_{i}}\right),X_{i+1}^{’}=\phi \left(X_{{\mathrm{out}}_{i+1}}\right)$$

where $X_{i}^{’}$ is the spatially downsampled shallow feature, $X_{i+1}^{’}$ is the deep feature after alignment, and $\phi \left(\cdot \right)$ denotes a 1 × 1 convolution, followed by batch normalization and ReLU.

**Figure 5:**
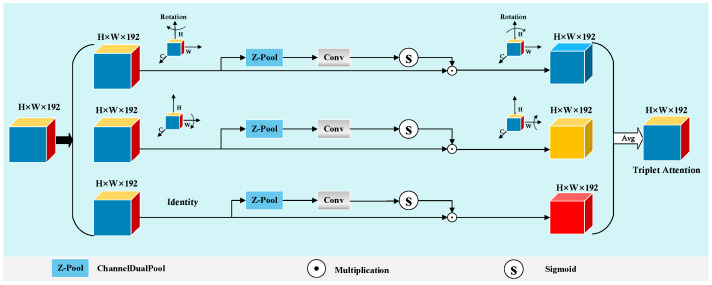
Triplet attention module.

The aligned features are then concatenated and passed through a triplet attention module to enhance critical spatial and channel-wise responses in lesion regions [42]. The structure of this process is illustrated in Figure 5, and the output is denoted as $F_{attn}$.

CoupleConv models lesion features across multiple scales by using a multi-branch convolutional block, with learnable fusion weights. Each branch applies a convolutional kernel with a different receptive field to extract semantic and structural cues at various scales. To improve the model’s computational efficiency and enhance representational diversity, grouped convolution (with group = 4) is employed within each branch, enabling parallel processing of channel groups and promoting functional decoupling. This process is shown in Equation (5).

(5) $$F_{\mathrm{multi}}=\sum_{k=1}^{K}\alpha_{k}\cdot {\mathrm{Conv}}_{k}\left(F_{\mathrm{attn}}\right)$$

where $F_{\mathrm{multi}}$ is the fused multi-scale feature, ${\mathrm{Conv}}_{k}$ represents the k-th convolutional branch, and $\alpha_{k}$ are dynamic weights obtained via Softmax, satisfying $\sum_{k=1}^{K}\alpha_{k}=1$.

Finally, the fused output is adaptively integrated with the aligned input features using learnable weights. This process is shown in Equation (6).

(6) $$F_{\mathrm{out}}=F_{\mathrm{multi}}+\omega_{1}\cdot X_{i}’+\omega_{2}\cdot {X’}_{i+1}$$

where $F_{\mathrm{out}}$ is the final output of the module, and $\omega_{1}$ and $\omega_{2}$ are Softmax-normalized weights, satisfying $\omega_{1}+\omega_{2}=1$.

### 3.4. Structure-Aware Guidance Module

**Figure 6:**
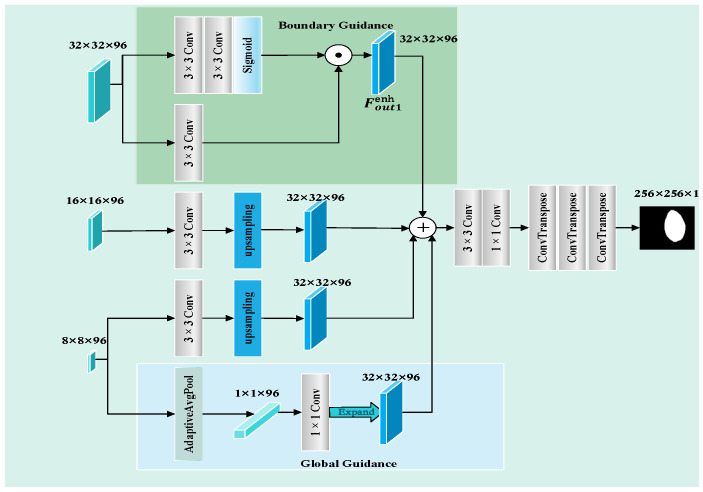
Structure-Aware Guidance Module (SAGM).

To address the limitations in structural perception during initial lesion localization, we propose the SAGM, as shown in Figure 6. This module is designed to integrate multi-level semantic features and enhance the generation of a structurally consistent coarse segmentation map through boundary guidance and global guidance. It receives as input three fused features, $F_{out1}$, $F_{out2}$, and $F_{out3}$, generated by the STFU, each integrating multi-scale information from different levels. It first applies three separate 3 × 3 convolutions to align channels and enhance nonlinearity, then upsamples $F_{out2}$ and $F_{out3}$ via bilinear interpolation to match the spatial resolution of $F_{out1}$.

A boundary detection branch then processes the shallow feature, $F_{out1}$, to generate an edge attention map, derived from cascaded convolutions and a sigmoid activation function. This map enhances the lesion edge regions within the shallow features, resulting in boundary-enhanced features. The module further incorporates global semantic information from the deepest feature, $F_{out3}$, extracting its global context vector via global average pooling, projecting it via a 1 × 1 convolution, and broadcasting it spatially to form the global guidance feature, $F_{glo}$. This process is shown in Equations (7) and (8).

(7) $$F_{out1}^{\mathrm{enh}}=F_{out1}\cdot A_{\mathrm{bnd}}$$

(8) $$F_{\mathrm{fused}}=\mathrm{Upsample}\left(F_{out2}\right)+\mathrm{Upsample}\left(F_{out3}\right)+F_{out1}^{\mathrm{enh}}+F_{\mathrm{glo}}$$

where $A_{\mathrm{bnd}}$ is the boundary attention map, $F_{\mathrm{glo}}$ the global guidance feature, $F_{out1}^{\mathrm{enh}}$ is the boundary-enhanced shallow feature, and $F_{\mathrm{fused}}$ is the fused feature from multiple sources.

The $F_{\mathrm{fused}}$ undergoes convolutional refinement and is progressively upsampled via transposed convolution layers to generate a 256 × 256 × 1 coarse segmentation map, which serves as structural guidance for the subsequent fine segmentation stage. SAGM employs a three-layer ConvTranspose architecture to gradually upsample the fused feature map from 32 × 32 to 256 × 256, aligning with the input image resolution. This progressive strategy is crucial for refining features through multiple convolutional stages, effectively mitigating checkerboard artifacts that are often introduced by abrupt upsampling, while better preserving lesion boundary details. Ablation studies based on the ISIC2018 dataset validate the necessity of this design: the reduction to two upsampling layers (32 × 32 → 128 × 128 → 256 × 256) led to a 0.82% decline in the mIoU and a 0.47% drop in the DSC, while a single-step upsampling directly to 256 × 256 resulted in more pronounced decreases of 1.35% in the mIoU and 0.79% in the DSC. These results confirm that the three-layer structure achieves an optimal trade-off between segmentation accuracy and computational efficiency.

### 3.5. Guided Multi-Scale Refiner

**Figure 7:**
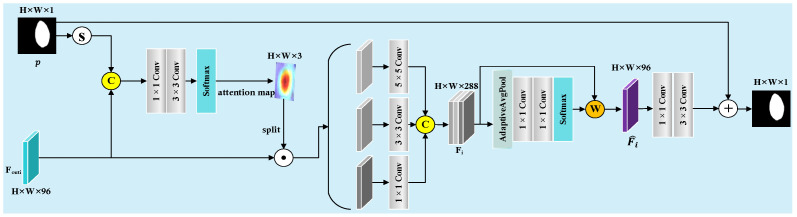
Guided Multi-Scale Refiner (GMSR).

While the existing decoder module can extract high-level semantic features, it lacks effective modeling and semantic alignment of fine-grained target details across multiple scales. In addition, the intermediate features in the network suffer from insufficient structure-aware enhancement during downsampling, which easily leads to blurred boundaries or unstable predictions. To address this, we designed a GMSR, as shown in Figure 7, to perform semantic-guided multi-scale modeling on decoding features at different scales and to improve the effectiveness and stability of training signals through a deep supervision mechanism.

The GMSR receives input from three intermediate features generated by the STFU and a coarse segmentation map from the SAGM. It processes the current-scale feature map $F_{outi}i\in \left(1,2,3\right)$, alongside the previous-stage prediction map p, to provide explicit semantic guidance. After sigmoid activation, the prediction map is concatenated with the feature map and passed through a set of convolutions to extract scale-related attention maps (three in total, corresponding to kernel sizes $\left[1,3,5\right]$). Each attention map modulates a corresponding convolution branch, enhancing the local response capability of features with different receptive fields.

Then, all of the scale-enhanced features are concatenated along the channel dimension to form the feature tensor. This process is shown in Equation (9).

(9) $$F_{i}=\mathrm{Concat}\left(F_{i}^{\left(1\right)},F_{i}^{\left(3\right)},F_{i}^{\left(5\right)}\right),F_{i}\in R^{H_{i}\times W_{i}\times 288}$$

where $F_{i}$ is the concatenated feature tensor, and $F_{i}^{\left(1\right)},F_{i}^{\left(3\right)},F_{i}^{\left(5\right)}$ are the enhanced features at different scales.

We apply global average pooling to extract the channel semantic vector, which is then passed through two layers of 1 × 1 convolutions to produce fusion weights for the three scale branches. This process is shown in Equation (10).

(10) $$W_{i}=\mathrm{Softmax}\left(W_{2}\cdot \sigma \left(W_{1}\cdot g_{i}\right)\right),W_{i}\in R^{1\times 1\times 3}$$

where $W_{i}$ is the fusion weight vector; $g_{i}$ is the channel semantic vector; $W_{1}$, $W_{2}$ are convolution parameters; and σ is the ReLU activation.

The final fused output feature is the weighted sum of the three scale-enhanced features. This process is shown in Equation (11).

(11) $$\hat{F_{i}}=\sum_{s=1}^{3}W_{i}^{\left(s\right)}\cdot F_{i}^{\left(s\right)},\hat{F_{i}}\in R^{H_{i}\times W_{i}\times 96}$$

where $\hat{F_{i}}$ is the fused feature map, $W_{i}^{\left(s\right)}$ is the weight of the s-th scale, and $F_{i}^{\left(s\right)}$ is the feature map of the s-th scale.

The aggregated features are fed into a two-layer convolutional structure to reduce the channel dimension and generate a single-channel output map. The GMSR produces a refined segmentation map, corresponding to the path before the summation node in the network architecture. It processes the current-scale feature map and the previous-stage prediction map to generate this single-channel output, which is connected to the upsampled previous-stage prediction map. This connection ensures coherent multi-scale feature refinement across different stages, enhancing the consistency and detail expression of the final prediction.

## 4. Experiments

### 4.1. Datasets

We used two ISIC datasets, ISIC2017 and ISIC2018, known for their large-scale skin image collections, for the segmentation tasks. The PH2 dataset was used for external validation of the ISIC2018-trained model to evaluate its generalization performance. Despite its smaller scale, the PH2 dataset’s high-quality annotations and melanoma samples make it ideal for testing segmentation models of high-risk lesions like melanoma, enabling the assessment of the model’s robustness across diverse clinical scenarios. Table 1 summarizes dataset usage, including sample divisions and purposes.

The ISIC and PH2 datasets may contain label noise, due to inconsistencies among annotators, low image contrast, or blurred lesion boundaries, which can adversely affect segmentation performance. To address this issue, we incorporated the DDBE module into SGNet to enhance the boundary features, thereby improving the model’s robustness to noisy annotations. Additionally, we applied data augmentation and normalization to reduce artifacts, such as hair interference. During the evaluation, multiple metrics were employed to ensure that the model maintained reliable performance in the presence of potential label inconsistencies. External validation based on the PH2 dataset further demonstrated the strong capability of SGNet in handling annotation noise.

### 4.2. Experimental Settings

The experimental settings are summarized in Table 2.

### 4.3. Objective Evaluation Indicators

The proposed method evaluates segmentation performance using six metrics: the mean Intersection over Union (mIoU), the Dice Similarity Coefficient (DSC), accuracy (ACC), sensitivity (SE), specificity (SP), and the Hausdorff Distance 95% (HD95). Based on the ISIC2017, ISIC2018, and PH2 datasets, the mIoU, DSC, ACC, SE, and SP are employed to compare model performance in skin lesion segmentation tasks, assessing the overlap and classification accuracy. To more precisely compare boundary delineation performance, the HD95 metric measures the 95th percentile of the maximum boundary distance between predicted and ground truth segmentations. By benchmarking against representative models, HD95 effectively highlights SGNet’s superior performance in complex skin lesion segmentation tasks, underscoring its value in evaluating boundary precision. The formulas for the evaluated metrics are detailed below.

(12) $$mIoU=\frac{TP}{FP+TP+FN}$$

(13) $$DSC=\frac{2TP}{FP+2TP+FN}$$

(14) $$ACC=\frac{TP+TN}{FP+TP+FN+TN}$$

(15) $$SE=\frac{TP}{TP+FN}$$

(16) $$SP=\frac{TN}{TN+FP}$$

where *FN* indicates false negative, *FP* indicates false positive, *TN* indicates true negative, and *TP* indicates true positive in the equations.

### 4.4. Comparison with State-of-the-Art Methods

#### 4.4.1. Quantitative Research

The experimental results based on the ISIC2017 dataset are presented in Table 3. UNet++, UNetV2, MFEFNet, VM-UNetV2, MHorUNet, and SwinUnet all achieved mIoU values above 80% and DSC values exceeding 89%. Compared to UNet++, UNetV2, MFEFNet, VM-UNetV2, MHorUNet, and SwinUnet, SGNet improved the mIoU values by 3.48%, 1.07%, 3.95%, 3.68%, 1.46%, and 2.74%, respectively. The corresponding DSC improvements were 1.92%, 0.58%, 2.12%, 2.02%, 0.66%, and 1.21%. Compared to all other methods, SGNet improved the mIoU by an average of 4.01% and the DSC by 2.28%, demonstrating superior segmentation performance based on the ISIC2017 dataset.

These results underscore SGNet’s ability to deliver superior segmentation performance and robustness. By effectively addressing challenges such as complex lesion boundaries and image noise, SGNet demonstrates a significant advancement over existing methods.

The experimental results based on the ISIC2018 dataset are presented in Table 4. UNetV2, MFEFNet, MHorUNet, SwinUnet, and VM-UNetV2 all achieved mIoU values above 80% and DSC values exceeding 89%. Compared to UNetV2, MFEFNet, MHorUNet, SwinUnet, and VM-UNetV2, SGNet improved the mIoU values by 1.76%, 1.74%, 1.38%, 1.23%, and 2.03%, respectively. The corresponding DSC improvements were 0.97%, 0.96%, 0.76%, 0.68%, and 1.12%. Compared to all of the other methods, SGNet improved the mIoU by an average of 2.87% and the DSC by 1.52%, demonstrating superior segmentation performance based on the ISIC2018 dataset.

Moreover, SGNet maintained competitive results in terms of accuracy, specificity, and sensitivity, demonstrating its ability to balance overall segmentation accuracy, specificity, and sensitivity. These results further validate the effectiveness of SGNet in addressing challenges such as complex lesion boundaries, variable contrast, and image noise.

To verify the generalization ability of the model, we used the PH2 dataset for external validation, where the model trained on the ISIC2018 dataset was directly applied to make predictions based on the PH2 dataset. The experimental results based on the PH2 dataset are presented in Table 5. UNetV2, MFEFNet, MHorUNet, VM-UNetV2, and SGNet all achieved mIoU values above 82% and DSC values exceeding 90%. Compared to UNetV2, MFEFNet, MHorUNet, and VM-UNetV2, SGNet improved the mIoU values by 1.95%, 1.36%, 0.78%, and 2.70%, respectively. The corresponding DSC improvements were 1.05%, 0.73%, 0.42%, and 1.41%. Compared to all the other methods, SGNet improved the mIoU by an average of 3.22% and the DSC by 1.73%, further demonstrating its superior generalization ability and segmentation performance based on unseen data.

Although SGNet significantly outperforms other state-of-the-art methods in terms of the mIoU and DSC, its SP is slightly lower than that of some competing models based on the ISIC2017 and ISIC2018 datasets. This outcome may be attributed to the model’s design emphasis on enhancing sensitivity to lesion regions through the STFU and GMSR modules. The STFU module adaptively integrates shallow texture features with deep semantic representations, enabling the model to better highlight ambiguous or low-contrast lesion areas. Meanwhile, the GMSR progressively refines the segmentation output under semantic guidance and deep supervision, enhancing the model’s responsiveness to fine boundaries and subtle structural cues. However, this focus on sensitivity and detailed boundary delineation may inadvertently increase the risk of over-segmenting non-lesion areas with similar visual characteristics. As a result, SGNet may generate more false positive predictions, leading to a slight decline in specificity.

**Figure 8:**
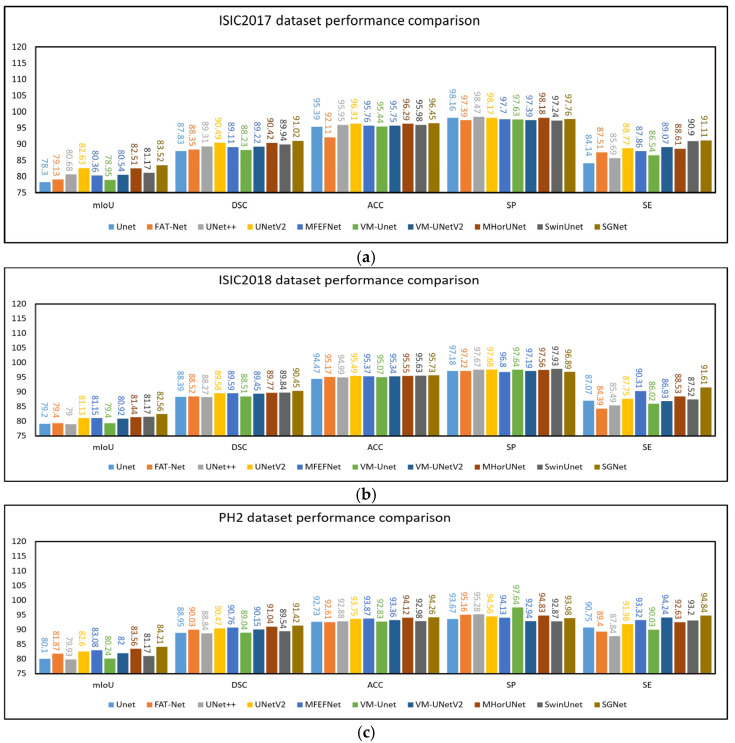
Performance of models based on different datasets: mIoU, DSC, ACC, SP, and SE. (**a**) ISIC2017 dataset performance comparison, (**b**) ISIC2018 dataset performance comparison, (**c**) PH2 dataset performance comparison.

The experimental results from SGNet based on the PH2 dataset demonstrate that it not only achieves excellent performance based on internal datasets, but also exhibits strong generalization ability during external validation. The design concept of SGNet, particularly its GMSR and DDBE, allows it to showcase outstanding adaptability and robustness in the challenging task of skin lesion image segmentation. In order to illustrate the above comparative experiments more intuitively, as shown in Figure 8a–c, this chapter presents the results of the comparative experiments based on the three datasets in the form of histograms.

SGNet significantly reduces the computational complexity, while maintaining high segmentation accuracy through the DDBE module. As shown in Table 6, SGNet has 30.26 M parameters, 7.92 G FLOPs, and an inference time of 0.012 s. Compared with other mainstream models, such as U-Net (32.08 M parameters, 16.59 G FLOPs, 0.008 s inference time), SwinUnet (41.39 M parameters, 11.37 G FLOPs, 0.214 s inference time), and UNet++ (47.20 M parameters, 65.58 G FLOPs, 0.112 s inference time), SGNet achieves an excellent balance between computational efficiency and segmentation performance. Compared to models with higher FLOPs, such as FAT-Net and UNet++, SGNet greatly reduces the computational cost, with only 7.92 G FLOPs required. Moreover, SGNet achieves a much shorter inference time of 0.012 s compared to SwinUnet, making it more suitable for real-time applications.

To further evaluate the performance of our proposed SGNet in regard to skin lesion segmentation tasks, we compared it with several representative models based on the ISIC2018 dataset. As shown in Table 7, SGNet achieved the best performance in regard to both the DSC and HD95, with a DSC of 90.19 ± 0.17 and a HD95 of 13.10 ± 0.25, significantly outperforming other competing methods. The *p*-values reported further highlight the superiority of SGNet in terms of DSC performance, indicating statistically significant improvements over all the baseline methods. These findings demonstrate that SGNet not only delivers superior overall segmentation accuracy, but also exhibits greater robustness and more precise boundary delineation.

#### 4.4.2. Qualitative Research

**Figure 9:**
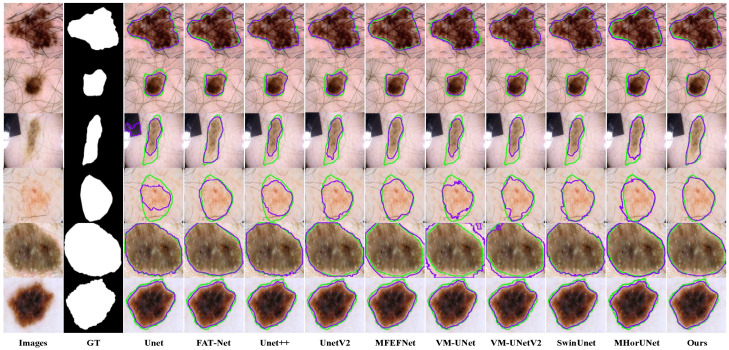
Segmentation performance based on the ISIC2017 dataset, where the green contour represents the ground truth segmentation, and the purple contour indicates the model’s predicted segmentation.

Existing models show clear limitations in segmenting the ISIC2017 dataset, as shown in Figure 9. The UNet architecture struggles to capture complex lesions, due to its simple design. This results in missed target regions and lower accuracy for detailed areas. FAT-Net demonstrates an insufficient capability in capturing fine boundaries of small lesion targets, resulting in frequent mis-segmentation. MFEFNet and VM-UNet exhibit misclassification in regard to low-contrast skin images, where lesion-background differentiation becomes challenging. Furthermore, VM-UNetV2, MHorUNet, and SwinUnet models are susceptible to hair interference, causing incomplete lesion segmentation or erroneous classification of hair as lesions or background areas. SGNet leverages the DDBE and STFU for robust segmentation. The DDBE employs a dual-domain approach, wherein its frequency branch applies an RFFT to transform feature maps, emphasizing boundary-related frequency components, while attenuating high-frequency noise associated with hair artifacts. This noise suppression mechanism ensures accurate lesion segmentation without misclassifying hair. The STFU complements the DDBE by adaptively fusing these enhanced boundary features with semantic information through a triplet attention mechanism and multi-branch convolution blocks. This synergy enables SGNet to maintain morphological consistency in hair-occluded regions.

**Figure 10:**
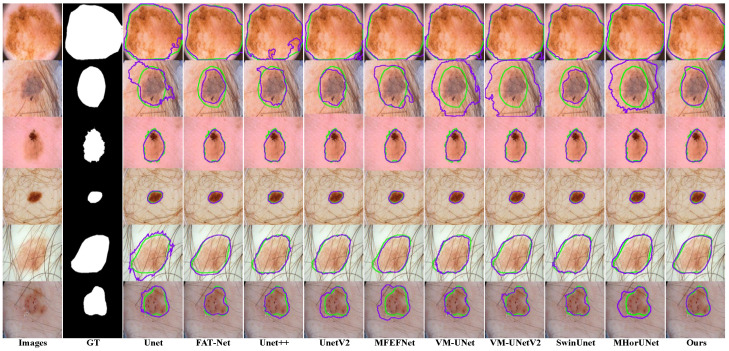
Segmentation performance based on the ISIC2018 dataset, where the green contour represents the ground truth segmentation, and the purple contour indicates the model’s predicted segmentation.

The segmentation results based on the ISIC2018 dataset, as shown in Figure 10, reveal distinct limitations among the existing models. UNet++ and UNetV2 involve improved architectures compared to their predecessors, but remain inadequate in regard to detecting subtle lesions. FAT-Net and MFEFNet generate false positive predictions in scenarios with complex textural backgrounds, while VM-UNetV2 and MHorUNet fail to fully mitigate partial missed detections under hair occlusion. Notably, SwinUnet demonstrates enhanced robustness against hair interference; however, its performance degrades in low-contrast regions, producing blurred boundaries. SGNet introduces the SAGM and GMSR, following the boundary enhancement and semantic fusion provided by the DDBE and STFU, to further improve segmentation accuracy. The SAGM integrates multi-level fused features to produce a coarse lesion localization map, which provides structural guidance for more accurate lesion detection. Building on this, the GMSR utilizes the guidance to progressively refine the lesion boundaries through the use of a multi-scale semantic attention mechanism. This two-stage process effectively restores precise contours and ensures morphological consistency.

**Figure 11:**
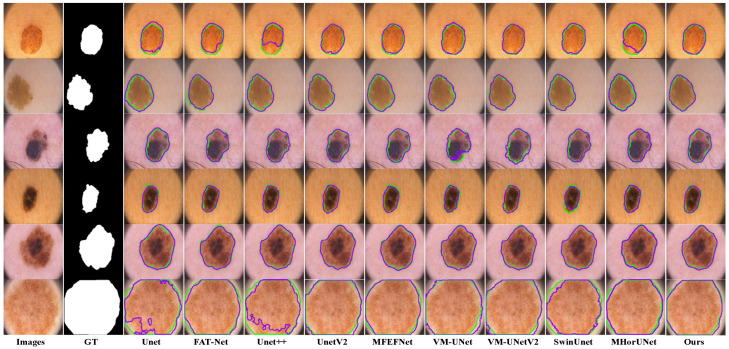
Segmentation performance based on the PH2 dataset, where the green contour represents the ground truth segmentation, and the purple contour indicates the model’s predicted segmentation.

The segmentation results based on the PH2 dataset, as shown in Figure 11, reveal distinct limitations among the existing models. UNet shows significant segmentation failures in the lesion regions, indicating weak generalization ability. FAT-Net and VM-UNet can roughly delineate the lesion regions, but with limited accuracy. MFEFNet, VM-UNetv2, MHorUNet, and SwinUnet can perform segmentation tasks successfully in most scenarios, with the segmentation results closely matching the ground truth, although their performance drops in low-contrast conditions. In contrast, SGNet accurately segments the lesion regions, unaffected by interference factors, and demonstrates strong generalization ability, outperforming the other networks.

**Figure 12:**
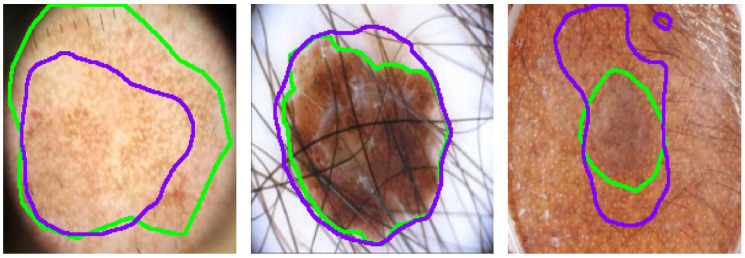
Analysis of SGNet failure cases involving complex image scenarios. where the green contour represents the ground truth segmentation, and the purple contour indicates the model’s predicted segmentation.

Despite SGNet’s robust performance, certain complex cases reveal limitations that require further investigation, as illustrated in Figure 12. In scenarios with severe hair interference, frequency-domain modeling struggles to fully suppress high-frequency artifacts, leading to partial mis-segmentation or edge confusion around occluded regions. Although the dual-domain design enhances boundary perception, densely distributed hair or noise-like patterns can introduce spectral components that resemble lesion textures, thereby misleading the segmentation process. Additionally, in regard to low-contrast backgrounds, where the intensity difference between the lesion and healthy skin is minimal, the model often exhibits blurred or uncertain boundary predictions. This is particularly evident in regard to lesions with fuzzy perimeters or irregular morphological structures, where both spatial cues and semantic priors are weak. These challenges complicate accurate lesion delineation.

### 4.5. Ablation Study

We conducted ablation experiments based on the ISIC2018 dataset to assess the contribution of each module in SGNet to the overall performance. The approach involved using VMamba as the baseline model and gradually adding the DDBE, STFU, GMSR, and SAGM in order to conduct a comparison.

The quantitative results in Table 8, where the “✓” symbol indicates the corresponding method applied in each model, show the performance of different module combinations based on the ISIC2018 dataset. Compared to the baseline model, Validation1, Validation2, and SGNet achieved relative improvements of 2.00%, 2.39%, and 2.76% in the mIoU, and 1.10%, 1.31%, and 2.15% in the DSC, respectively. These results confirm the effectiveness of the individual modules, demonstrating their ability to significantly enhance the network’s segmentation performance and overall predictive capability.

**Figure 13:**
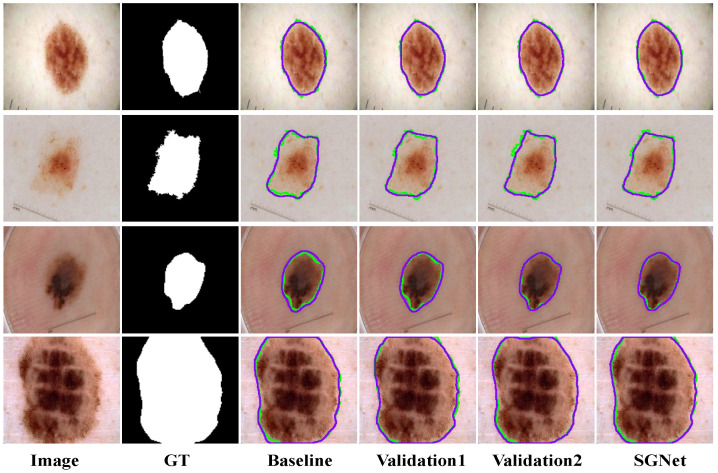
Ablation study visualization based on ISIC2018. The green contour lines represent the ground truth, and the purple contour lines represent the model’s predicted segmentation.

The influence of different modules on the model’s performance based on the ISIC2018 dataset is shown in Figure 13. The baseline model, based on the VMamba encoder, effectively extracts multi-level features; however, due to the lack of constraints on low-level boundary information, its representational capability at lesion edges is limited. To address this issue, the DDBE is introduced, which jointly models spatial and frequency domains to suppress noise interference and enhance the boundary representation of low-level features. Despite this improvement, cross-level feature fusion remains insufficient, restricting the model’s ability to fully capture structural details. To mitigate this, the STFU is incorporated to adaptively fuse features across different resolutions, thereby improving the collaborative modeling of the spatial structure and semantic context. Subsequently, the SAGM generates coarse lesion localization maps that provide global semantic guidance for subsequent segmentation. Finally, the GMSR employs a guided multi-scale semantic attention mechanism to progressively refine high-resolution features, significantly enhancing the model’s representation of lesion boundaries and local details.

## 5. Conclusions

This paper presents SGNet, a novel network for skin lesion segmentation, designed to address limitations, such as insufficient local feature extraction, inefficient multi-scale feature fusion, and inaccurate boundary localization. SGNet integrates five specialized modules to enable the effective capturing of local details, the coherent integration of semantic and spatial information, and accurate delineation of irregular lesion boundaries. Extensive experiments based on the ISIC2017, ISIC2018, and PH2 datasets demonstrate the superior segmentation performance of SGNet, which achieves an average improvement of 3.30% in the mIoU and 1.77% in the DSC compared to state-of-the-art methods. Although SGNet achieves outstanding overall performance in skin lesion segmentation, its specificity is slightly lower than that of some competing models, and its segmentation accuracy may be challenged in cases with low contrast or heavy artifact interference. These limitations suggest that further refinement is needed to improve the model’s robustness in more complex real-world scenarios. In future work, we aim to enhance SGNet to accommodate a broader range of medical image analysis tasks. We will explore cross-modal or multi-modal information fusion strategies to improve the model’s generalization capability under diverse data conditions. These efforts will further advance SGNet toward becoming a more comprehensive and intelligent system for computer-aided diagnosis of skin lesions.

## Figures and Tables

**Table 1:** The usage status of the datasets.

Dataset	Images	Input Size	Train	Valid	Test
ISIC2017	2000	256 × 256	1250	150	600
ISIC2018	2594	256 × 256	1620	195	779
PH2	200	256 × 256	0	0	200

**Table 2:** Experimental settings.

Parameter	Setting
Hardware	NVIDIA GeForce GTX 3090 GPU
Framework	PyTorch 2.0.1
Data Augmentation	Random flipping, random rotation
Loss Function	BCE-Dice
Batch Size	16
Optimizer	AdamW (initial learning rate: 1 × 10^−3^)
Scheduler	CosineAnnealingLR
Training Duration	100 epochs

**Table 3:** ISIC2017 dataset performance comparison.

Dataset	Model	mIoU	DSC	ACC	SP	SE
	UNet [8]	78.30	87.83	95.39	98.16	84.14
	FAT-Net [17]	79.13	88.35	92.11	97.39	87.51
	UNet++ [16]	80.68	89.31	95.95	98.47	85.69
	UNetV2 [43]	82.63	90.49	96.31	98.17	88.77
	MFEFNet [14]	80.36	89.11	95.76	97.70	87.86
ISIC2017	VM-UNet [35]	78.95	88.23	95.44	97.63	86.54
	VM-UNetV2 [36]	80.54	89.22	95.75	97.39	89.07
	MHorUNet [25]	82.51	90.42	96.29	98.18	88.61
	SwinUnet [15]	81.17	89.94	95.98	97.24	90.90
	SGNet	83.52	91.02	96.45	97.76	91.11

**Table 4:** ISIC2018 dataset performance comparison.

Dataset	Model	mIoU	DSC	ACC	SP	SE
	UNet [8]	79.20	88.39	94.47	97.18	87.07
	FAT-Net [17]	79.40	88.52	95.17	97.22	84.39
	UNet++ [16]	79.00	88.27	94.99	97.67	85.49
	UNetV2 [43]	81.13	89.58	95.49	97.68	87.75
	MFEFNet [14]	81.15	89.59	95.37	96.80	90.31
ISIC2018	VM-UNet [35]	79.40	88.51	95.07	97.64	86.02
	VM-UNetV2 [36]	80.92	89.45	95.34	97.19	86.93
	MHorUNet [25]	81.44	89.77	95.55	97.56	88.53
	SwinUnet [15]	81.56	89.84	95.63	97.93	87.52
	SGNet	82.56	90.45	95.73	96.89	91.61

**Table 5:** PH2 dataset performance comparison.

Dataset	Model	mIoU	DSC	ACC	SP	SE
	UNet [8]	80.10	88.95	92.73	93.67	90.75
	FAT-Net [17]	81.87	90.03	92.61	95.16	89.40
	UNet++ [16]	79.93	88.84	92.88	95.28	87.84
	UNetV2 [43]	82.60	90.47	93.75	94.56	91.98
	MFEFNet [14]	83.08	90.76	93.87	94.13	93.32
PH2	VM-UNet [35]	80.24	89.04	92.83	97.64	90.03
	VM-UNetV2 [36]	82.00	90.15	93.36	92.94	94.24
	MHorUNet [25]	83.56	91.04	94.12	94.83	92.63
	SwinUnet [15]	81.06	89.54	92.98	92.87	93.20
	SGNet	84.21	91.42	94.26	93.98	94.84

**Table 6:** Comparison of model computational efficiency.

Model	Parameter	FLOPs	Inference Time
UNet [8]	32.08 M	16.59 G	0.008 s
FAT-Net [17]	28.76 M	49.39 G	0.035 s
UNet++ [16]	47.20 M	65.58 G	0.112 s
UNetV2 [43]	25.15 M	43.20 G	0.048 s
MFEFNet [14]	48.36 M	14.03 G	0.019 s
VM-UNet [35]	22.03 M	4.11 G	0.009 s
VM-UNetV2 [36]	22.77 M	4.40 G	0.010 s
MHorUNet [25]	4.96 M	9.19 G	0.038 s
SwinUnet [15]	41.39 M	11.37 G	0.214 s
SGNet	30.26 M	7.92 G	0.012 s

**Table 7:** Comparison results based on ISIC2018 dataset for skin lesion segmentation.

Dataset	Model	DSC	HD95	*p*-Value(DSC)
	nnUNet [18]	88.3 ± 0.19	24.10 ± 0.32	0.0013
	SwinUnet [15]	89.61 ± 0.16	19.00 ± 0.26	0.0181
ISIC2018	VM-UNetV2 [36]	89.29 ± 0.12	18.95 ± 0.22	0.0076
	SGNet	90.19 ± 0.17	13.10 ± 0.25	

**Table 8:** The performance contribution of different modules based on the ISIC2018 dataset.

Model	DDBE	STFU	GMSR + SAGM	mIoU	DSC
Baseline				80.34	89.10
Validation1	✓			81.95	90.08
Validation2	✓	✓		82.26	90.27
SGNet	✓	✓	✓	82.56	91.02

## Data Availability

The data are contained within the article.
